# Why are listeners hindered by talker variability?

**DOI:** 10.3758/s13423-023-02355-6

**Published:** 2023-08-14

**Authors:** Sahil Luthra

**Affiliations:** 1 Department of Psychology, Carnegie Mellon University, 5000 Forbes Ave, Pittsburgh, PA 15213, USA

**Keywords:** Talker variability, Normalization, Attention, Auditory streaming, Speech perception

## Abstract

Though listeners readily recognize speech from a variety of talkers, accommodating talker variability comes at a cost: Myriad studies have shown that listeners are slower to recognize a spoken word when there is talker variability compared with when talker is held constant. This review focuses on two possible theoretical mechanisms for the emergence of these processing penalties. One view is that multitalker processing costs arise through a resource-demanding talker accommodation process, wherein listeners compare sensory representations against hypothesized perceptual candidates and error signals are used to adjust the acoustic-to-phonetic mapping (an active control process known as *contextual tuning*). An alternative proposal is that these processing costs arise because talker changes involve salient stimulus-level discontinuities that disrupt *auditory attention*. Some recent data suggest that multitalker processing costs may be driven by both mechanisms operating over different time scales. Fully evaluating this claim requires a foundational understanding of both talker accommodation and auditory streaming; this article provides a primer on each literature and also reviews several studies that have observed multitalker processing costs. The review closes by underscoring a need for comprehensive theories of speech perception that better integrate auditory attention and by highlighting important considerations for future research in this area.

## Introduction

The ease with which listeners recognize a talker’s intended speech sounds belies the computational complexity of speech perception. Even within a single dialect, there is a considerable degree of acoustic variability in how speech sounds are produced, with acoustic details varying as a function of factors like phonetic context (Liberman et al., 1967), speaking rate (Miller, 1981), and talker (Hillenbrand et al., 1995; Peterson & Barney, 1952). Furthermore, the acoustic-phonetic details of the speech signal are shaped by a variety of socio-indexical features, such as age, sex, and gender (Lee et al., 1999; Munson & Babel, 2019). Despite this variability, listeners readily achieve stable percepts of the intended phonetic categories—that is, listeners achieve *phonetic constancy* despite a lack of invariance between acoustic cues and phonetic percepts (e.g., Liberman et al., 1957).

Contending with variability, however, can come at a cost. In the case of talker variability, listeners are typically slower or less efficient in processing speech when there is talker variability compared with when talker is held constant. Such *multitalker processing costs* have been observed in a variety of studies, though the vast majority of them have used speeded word monitoring (Heald & Nusbaum, 2014; Magnuson & Nusbaum, 2007; Nusbaum & Morin, 1992; Wong et al., 2004) or speeded word classification (Choi et al., 2018; Mullennix & Pisoni, 1990) paradigms. Though accuracy tends to be high in the face of talker variability, listeners are generally slower (and sometimes less accurate; e.g., Creelman, 1957; Goldinger et al., 1991; Kapadia et al., 2023; Lim et al., 2021; Nusbaum & Morin, 1992; Sommers, 1996) when consecutive stimuli are spoken by different talkers compared with when stimuli are spoken by a single talker. Neuroimaging studies have also revealed differences in brain responses when there is talker variability compared with when talker is held constant, both in fMRI (Perrachione et al., 2016; Wong et al., 2004), and EEG (Uddin et al., 2020) data.

Historically, such findings have been taken as evidence that speech perception requires adjusting for acoustic-phonetic differences between talkers and that there is some processing penalty associated with performing this adjustment (Mullennix et al., 1989). In other words, multitalker processing costs arise through the perceptual accommodation of talker-phonetic variability. The term *talker normalization* is typically used to refer to the process by which listeners bring the surface form of an acoustic production into registration with their internal phonetic categories (Joos, 1948; Nearey, 1989; Pisoni, 1997).

**Fig. 1:**
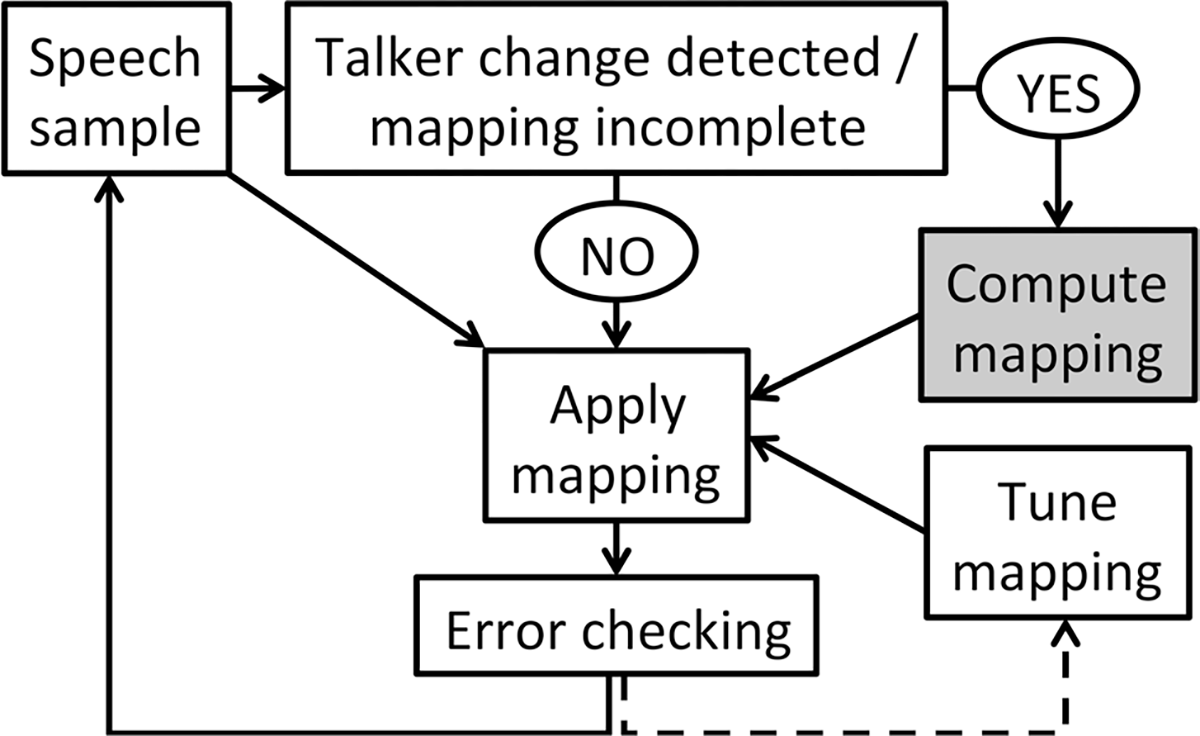
A schematic of the *contextual tuning* theory, which has been used to account for multitalker processing costs. On this view, whenever listeners detect that the current mapping between acoustics and phonetic categories is incomplete (e.g., when there is an overt change in talker), a resource-demanding mapping computation stage (gray box) is initiated. Because the direction of information flow in this model is dependent on the outcome of previous computations, this is an example of an *active control* model. Source: Magnuson (2018)

While researchers have proposed multiple specific mechanisms by which talker normalization might be achieved, most approaches share the assumption that normalization proceeds in a passive, automatic fashion (Weatherholtz & Jaeger, 2016). However, some researchers have suggested that the accommodation of talker variability requires an active control process, whereby cognitive mechanisms are used to adaptively adjust the mapping between acoustics and phonetic categories (Fig. 1). Critically, these researchers have suggested that multitalker processing costs arise through a resource-demanding mapping computation specifically (Magnuson & Nusbaum, 2007), rather than reflecting a passive normalization process; this view is most strongly supported by evidence that multitalker processing costs are exacerbated under increased working memory load (Nusbaum & Morin, 1992). Importantly, the term *talker normalization* does not inherently imply a passive mechanism, but because of the widespread view that normalization is achieved through automatic, low-level processes (see Weatherholtz & Jaeger, 2016, for discussion), this review favors the phrase *talker accommodation* as an umbrella term for both passive normalization and active control mechanisms through which listeners might cope with talker-phonetic variability. In the following section, I provide an overview of the literature on talker accommodation and review some of the evidence consistent with an active control mechanism (specifically, the *contextual tuning* model) that has been invoked to explain multitalker processing costs.

A prominent alternative hypothesis holds that multitalker processing costs arise not due to a talker accommodation process but rather because of a disruption in top-down attention by a discontinuity in auditory streaming (Lim et al., 2019); the third section, “A Role for Auditory Attention,” provides a primer on the auditory streaming literature that motivates this *auditory attention hypothesis* and considers some of the recent evidence consistent with this view.

As a brief aside, it is also worth noting that other hypotheses have been put forth that might explain the emergence of multitalker processing costs, though a thorough discussion of all these accounts is beyond the scope of this review. First, under an *episodic* view, listeners retain perceptually rich episodes of words that they hear rather than abstracting away from talker-specific detail (Goldinger, 1998); more recently encountered talkers are represented more strongly in memory, leading to a performance advantage when talker is held constant (and one can compare the incoming speech to highly active representations from that same talker) compared with when talker is variable (and the relevant episodes may not be strongly active). By contrast, the *efficient coding* hypothesis holds that perception is facilitated when input is highly structured, leading to performance benefits in single-talker conditions because the input is less variable as compared with mixed-talker conditions; consistent with this view, multitalker processing costs are relatively small when there is little F0 variability between talkers as well as when talker changes occur relatively infrequently (Stilp & Theodore, 2020). Finally, multitalker processing costs could also be explained through *Bayesian belief-updating accounts* (Kleinschmidt & Jaeger, 2015), which hold that listeners maintain probabilistic beliefs (generative models) of how acoustics map to phonetic categories, and these beliefs are often talker-specific; there may be some cost associated with switching generative models following a talker change, explaining the emergence of multitalker processing costs. Notably, these accounts are not necessarily mutually exclusive and may indeed be functionally equivalent in some cases. For instance, the mapping computation stage of the contextual tuning model (Fig. 1) might be functionally equivalent to switching one’s generative model, such that the contextual tuning theory might be fully compatible with a Bayesian belief-updating account. Because the literature on multitalker processing costs has primarily considered the contextual tuning and auditory attention hypotheses, this review focuses on those two possible mechanisms specifically.

Recently, Choi et al. (2022) proposed that multitalker processing costs might be driven by *both* talker accommodation and auditory attention mechanisms, with the two mechanisms operating over different time scales. To assess the degree to which this might be the case, it is critical that researchers examining multitalker processing costs have a foundational understanding of both mechanisms. However, thus far, the literatures on talker accommodation and auditory streaming have made relatively little contact with each other. The aim of this review is to provide a primer on each literature, thereby allowing researchers to better consider how these processing penalties might be driven by talker accommodation and auditory attention mechanisms. In the section titled “What Drives Multitalker Processing Costs?,” I review recent data on the emergence of multitalker processing costs, and in the section titled “Toward an Integrated Account of Auditory Attention and Talker Accommodation,” I highlight important questions for future research investigating the degree to which each of these mechanisms underlies the processing costs associated with talker variability.

## The perceptual accommodation of talker variability

### Normalization mechanisms

Listeners encounter a great deal of acoustic variability between talkers. To illustrate, consider that different vowels (the /i/ in *heed,* the /ɪ/ in *hid*, etc.) can be distinguished based on their location in an acoustic space defined by the first and second formants, denoted as F1 and F2, respectively; F1 and F2 are two frequency bands in which acoustic energy is highly concentrated as a result of the resonant frequencies of the vocal tract, and they vary with vocal tract configurations such as tongue and lip positions. Critically, however, formant values are highly variable, both within a single talker and between different talkers. If one plots several talkers’ productions of a set of vowels in F1 × F2 space, there is considerable overlap between adjacent categories, such that one talker’s production of /i/ might have first and second formant values identical to those from another talker’s production of /ɪ/ (Hillenbrand et al., 1995; Peterson & Barney, 1952). Despite this acoustic variability, listeners typically exhibit near-ceiling accuracy in their classification of the talkers’ intended vowel productions.

Joos (1948) proposed a mechanism by which listeners might compensate for this between-talker variability. To accurately identify vowels, listeners would simply need samples of a talker’s point vowels (i.e., vowels like /æ/, /i/, and /u/, which occupy the most extreme regions of F1 × F2 acoustic space). Knowing where these most extreme vowels reside in acoustic space would allow a listener to determine where the other vowels should be placed in a normalized acoustic space. Algorithmically, this might be achieved via a simple scaling transformation (e.g., Gerstman, 1968), and accurate identification of vowels would be achieved based on their location in a normalized F1 × F2 space. To correctly identify a talker’s vowels, a listener would only need a sample of the talker’s speech that provided sufficient information about the resonant frequencies of the point vowels; a common greeting such as “How do you do?” might serve this purpose (Joos, 1948). Through such a rescaling process, listeners who had even a basic degree of familiarity with a talker’s vowel space would be able to rescale that talker’s subsequent vowels, allowing them to cope with between-talker acoustic-phonetic variability.

The precise mechanism proposed by Joos (1948) has since been challenged. Evidence shows that listeners are able to identify relatively short utterances from unfamiliar talkers with high accuracy, even without prior exposure to point vowels (Shankweiler et al., 1977), and prior exposure to a talker’s point vowels does not always reduce error rates in subsequent vowel recognition (Verbrugge et al., 1976). Nonetheless, the hypothesis of a normalization procedure remains influential, with myriad specific mechanisms proposed. A core principle across different normalization hypotheses is the notion that the acoustic variability between talkers is not random—rather, there is *structure* to between-talker variability, and it is this structure that listeners leverage in order to infer the phonetic identity of a speech segment (Kleinschmidt, 2019). Such a view contradicts the way that talker normalization has been characterized by some researchers (e.g., Palmeri et al., 1993; Pisoni, 1997), who have argued that the word “normalization” inherently involves discarding irrelevant information about talker.^1^ However, accommodating talker variability does not require losing information about the speaker (Magnuson & Nusbaum, 2007). Instead, it can and should be conceptualized as a process that capitalizes on the systematic ways in which acoustic productions differ across talkers.

Researchers have made a distinction between two theoretically distinct forms of normalization. *Intrinsic normalization* is driven by information within the to-be-normalized speech signal (Ainsworth, 1975; also referred to as *structural estimation* by Nusbaum & Morin, 1992) and might be achieved by scaling vowel formants relative to each other (Johnson, 2008; Syrdal & Gopal, 1986; for a historical review, see Miller, 1989) as well as by utilizing dynamic cues immediately adjacent to the vowel nucleus (Jenkins et al., 1994; Sussman et al., 1991, 1993; Sussman & Shore, 1996). Additionally, *extrinsic normalization* (Ainsworth, 1975) is a mechanism wherein listeners use preceding context to guide the interpretation of subsequent auditory information (for a recent review, see Stilp, 2020). For instance, several studies have shown that the spectrotemporal information in a carrier sentence (e.g., *Please say what this word is*) can influence the interpretation of a subsequent auditory target (e.g., influencing whether a segment is identified as *bit* or *bet*; Bosker, 2018; Ladefoged & Broadbent, 1957; Sjerps et al., 2018). Notably, some work has even shown that the long-term average spectrum of a non-speech auditory context (e.g., a sequence of sine-wave tones) can influence identification of a subsequent speech target, raising the possibility that extrinsic normalization may be supported by general auditory mechanisms, rather than speech-specific ones (Laing et al., 2012).

Empirical evidence supports the use of both intrinsic and extrinsic normalization in the accommodation of between-talker acoustic-phonetic variability. For instance, listeners are relatively accurate in identifying speech even when the vowel nucleus has been replaced with silence, so long as they still have access to both the vowel onset and offset; this is the case even when the vowel onset and offset are produced by different talkers, suggesting that listeners can leverage talker-independent cues within a vowel to identify a phoneme (Jenkins et al., 1994). Furthermore, even minimal exposure to a talker’s speech—for instance, just hearing isolated productions of that talker’s /i/ vowel—can boost recognition accuracy for subsequent speech (Morton et al., 2015). This suggests that early exposure to a talker’s vowel space provides listeners with a perceptual baseline against which to evaluate subsequent speech. Importantly, intrinsic normalization and extrinsic normalization need not be viewed as alternatives; rather, normalization is likely guided by both intrinsic and extrinsic mechanisms (Nearey, 1989).

Nevertheless, normalization may be insufficient for fully accommodating talker variability. A recent corpus analysis found that after applying a simple normalization transformation (*z*-scoring F1 and F2; Lobanov, 1971), there was still a considerable degree of between-talker variability, and even after normalization, socio-indexical factors like gender provided additional information about the phonetic identity of a speech sound (Kleinschmidt, 2019). Such a result suggests that listeners need to do more than simply normalize the speech signal in order to accommodate talker differences (though it does not mean that normalization mechanisms are not at play).

### Active control mechanisms

Researchers have also argued that a passive normalization mechanism is insufficient for accommodating all between-talker variability on theoretical grounds (Nusbaum & Magnuson, 1997). In a passive perceptual system, a given input is always associated with an invariant output, even if a large number of transformations may potentially be involved in mapping from input to output (Nusbaum & Schwab, 1986). In such a system, information only flows in one direction, with no feedback (i.e., it is an open-loop system). However, the fact that different talkers produce their speech sounds differently means that many different acoustic productions can correspond to one phonetic category (a many-to-one mapping). Furthermore, the same acoustic signal can also map onto different phonetic categories (a one-to-many mapping) depending on factors such as phonological context (Liberman et al., 1952; Mann & Repp, 1981) or even a listener’s expectations about talker gender (Johnson et al., 1999). As such, the transformation between acoustics and phonetic categories involves a many-to-many mapping, and therefore speech perception inherently constitutes a nondeterministic process (Nusbaum & Magnuson, 1997). Because of the lack of invariance between input and output in speech perception, a passive normalization mechanism alone cannot be used to solve the speech perception problem.

Instead, selecting between possible interpretations of a production (e.g., knowing whether the acoustic-phonetic information cues /ε/ or /æ/) requires an *active control* system (Nusbaum & Schwab, 1986), whereby a given input can map to multiple possible outputs. An active control system involves a comparison between a representation of an input and hypotheses about what elicited the input; there need not be top-down feedback, but what is critical is that information flow depends on previous computations, allowing for error-based adjustments to information processing. In an active control model of speech perception, difficulties mapping parsed phonetic categories to words could drive implicit adjustments to the mapping; for instance, a parse of *Weckud Wetch of the Wast* could indicate the need to shift the acoustic-phonetic mapping to yield a parse of *Wicked Witch of the West* (Maye et al., 2008). Because a passive system involves mapping from an input to a fixed response, passive processing can proceed automatically. However, because an active control system involves contending with a many-to-many mapping and making adjustments to processing based on previous computations, *an active control system requires the use of (and is constrained by the availability of) cognitive resources*.

Thus, while passive normalization mechanisms could be useful for accommodating several types of acoustic variability, listeners also need to adjust the mapping between acoustic cues and phonetic categories in order to accommodate acoustic-phonetic variability. Normalization and active control need not be thought of as mutually exclusive options; instead, they can be seen as complementary mechanisms for accommodating talker variability. (Such a view parallels the way other forms of normalization have been described; for example, for recent evidence that rate normalization and cue-category adjustments are dissociable processes, with normalization occurring during the encoding of auditory information and cue-category adjustments occurring at a higher stage of processing, see Lehet & Holt, 2020.)

### A contextual tuning view of multitalker processing costs

As outlined in Fig. 1, the *contextual tuning* theory proposed by Nusbaum and Magnuson (1997) holds that when listeners detect that the existing mapping between acoustics and phonetics is no longer appropriate, they must compute the correct mapping between the acoustic signal and their perceptual categories (or possibly, retrieve an existing mapping from memory; Magnuson et al., 2021). Though such an adjustment could in principle be achieved by passive (extrinsic) normalization mechanisms, the active control view of multitalker processing costs holds this mapping adjustment (gray box in Fig. 1) is a resource-demanding cognitive process, and it is in this way that talker variability elicits a multitalker processing cost. Note that in this contextual tuning model, the flow of information is dependent on the outcome of previous stages; for instance, the “compute mapping” stage might never be initiated if the current mapping is appropriate. As such, the contextual tuning model is classified as a specific example of an active control model.

The idea that multitalker processing costs arise through a resource-demanding cognitive process has been most strongly supported by evidence that multitalker processing costs are modulated by demands on working memory. Nusbaum and Morin (1992) found that listeners were slower in a syllable monitoring task when there was talker variability compared with when talker was held constant; furthermore, this multitalker processing cost was exacerbated when listeners had to hold three two-digit numbers in memory as compared with when they only had to hold one such number in working memory. Nusbaum and Magnuson (1997) interpreted this finding as evidence that multitalker processing costs arise through a resource-demanding talker accommodation process. They reasoned that the task of holding several digits in memory also requires cognitive resources, thereby reducing the amount of cognitive resources available to accommodate talker differences and thus evoking a larger multitalker processing cost.

Some additional evidence for the view that multitalker processing costs emerge through an active control process came from a study by Magnuson and Nusbaum (2007). In an active control loop, processing is permeable to high-level expectations; as such, the emergence of a multitalker processing cost could be modulated by a listener’s expectations about how many talkers they will hear. Listeners in this study heard two sets of synthetic stimuli, one produced with an average fundamental frequency (F0) of 150 Hz and one with an average F0 of 160 Hz. One group of listeners was told that these stimuli corresponded to a single synthetic talker with variable pitch and heard a monologue with variable F0. A second group of listeners was told that these stimuli were being used to simulate two different talkers, and their expectations were reinforced through a dialogue between two talkers with different F0. Listeners then completed a speeded monitoring task. Magnuson and Nusbaum observed a multitalker processing cost in the group that expected two voices, but not in the group that expected one voice, consistent with an active control architecture. Notably, however, a recent well-powered, preregistered study failed to replicate this finding; in that replication attempt, multitalker processing costs were not observed in either group of listeners (Luthra et al., 2021). Nonetheless, expectations about talker identity have been shown to influence processing in other paradigms; for instance, one study found that listeners were less likely to notice a change in talker during a telephone conversation when they were not explicitly monitoring for one (Fenn et al., 2011), and another found that classification of ambiguous vowels was influenced by a listener’s expectations about talker gender (Johnson et al., 1999).

In recent years, other researchers have proposed an alternative mechanism through which multitalker processing penalties might arise (Lim et al., 2019). Instead of reflecting a resource-demanding mapping adjustment process, multitalker processing costs may simply reflect a disruption in auditory selective attention. On this account, a change in talker, signaled by discontinuities in the acoustic features of the speech, is heard as a new stream, eliciting an involuntary reorienting of attention and thereby incurring a multitalker processing cost. In the next section, I provide a primer on auditory scene analysis and review some recent evidence consistent with the auditory attention view of multitalker processing costs.

## A role for auditory attention

### Foundational principles of auditory streaming

A related but theoretically distinct issue from the lack of invariance problem is the issue of how listeners selectively attend to relevant auditory information during speech perception. Researchers often refer to this as the *cocktail party* problem (Cherry, 1953), as a cocktail party requires a listener to attend to speech from one physical source (namely, the person one is conversing with) and ignore competing sources of auditory input (nearby individuals engaged in other conversations). Despite the challenges of cocktail party situations, most listeners can readily attend to a target talker in multitalker environments, as evidenced by both behavioral and neural data. Salient spectrotemporal features of an attended talker can be recovered from neural activity measured from surface electrodes placed on auditory cortex, and spectrograms reconstructed from the cortical activity in multitalker listening conditions show strong resemblances to single-talker spectrograms of the attended talker, suggesting robust and selective encoding of an attended talker in cocktail party environments (Mesgarani & Chang, 2012). While a thorough review of selective attention is beyond the scope of this paper, there are several excellent reviews (Bee & Micheyl, 2008; Bregman, 1990; Bronkhorst, 2015; Fritz et al., 2007; Holmes et al., 2021; Shinn-Cunningham, 2008; Shinn-Cunningham et al., 2017; Snyder & Elhilali, 2017) that complement the overview provided in this article.

In a landmark book on auditory scene analysis, Bregman (1990) described the importance of *simultaneous integration*—namely, the process of determining, at each point in time, which simultaneously occurring parts of the frequency spectrum should be perceptually grouped. The nearly synonymous terms *stream segregation* and *auditory object formation* are often used to describe this process, as simultaneous integration involves segregating the stream of interest from the background, allowing listeners to group together the components of the auditory signal that come from the same auditory source (Bronkhorst, 2015; Shinn-Cunningham et al., 2017). Distinct from simultaneous integration is *sequential integration*, which refers to the process of linking across time, or *streaming*, the target parts of the auditory signal; the term *auditory object selection* is also often used here. While the distinction between simultaneous and sequential integration is useful, it is important to note that these should not be thought of as entirely separable processes, occurring one after the other; rather, it is useful to think of segregation and streaming as interdependent processes that influence each other and are generally both at play during auditory scene analysis (Shinn-Cunningham et al., 2017).

As a simple illustration of segregation and streaming, consider a series of tones at two different frequencies occurring in an ABA-ABA- pattern. Such a pattern may be perceived as coming from a single source (i.e., an *integrated* percept that sounds like galloping) or as coming from two sources (i.e., two *segregated* streams, one that sounds like A-A-A-A- and one that sounds like B---B---). Depending on factors such as the timing and frequency separation of the A and B tones, they may be integrated (for slower rates and smaller frequency separations), segregated (for faster rates and larger frequency separations), or ambiguous (Bregman et al., 2000). Streaming can also be influenced by recent experience; for instance, the frequency separation of a preceding context has been shown to affect the likelihood of streaming for a subsequent ambiguous A-B pattern (Snyder et al., 2008; Yerkes et al., 2019).

For ambiguous A-B patterns, streaming builds up over time: Listeners will tend to hear the integrated percept initially, but by the end of a sequence, they are likely to hear two segregated streams (Snyder et al., 2006). Notably, the buildup of streaming can be reset by discontinuities (e.g., silent gaps) in the sequence (Cusack et al., 2004), suggesting that streaming is at least partly governed by stimulus-level properties. However, listeners have also been shown to be able to exert some degree of voluntary control over whether they hear an integrated stream or two segregated streams in these paradigms; strikingly, when listeners attempt to exert volitional control over how they perceive the streams (e.g., when they intend to hear two segregated streams), the neural responses in auditory cortex resemble the neural responses elicited when listeners do not attempt to shape their perception but happen to perceive the tones in that way (e.g., as segregated), suggesting a perceptual effect rather than a response bias (Billig et al., 2018).

Some research has suggested a role for attention in the buildup of streaming, with evidence that the degree of streaming is modulated by whether listeners are initially directed to attend to a competing auditory stimulus (Carlyon et al., 2001; Cusack et al., 2004). Consistent with this proposal, one electrophysiological study observed an event-related potential from 150 to 250 ms that appeared to correlate with streaming in that it (a) built in size as listeners heard more tones and (b) was larger when listeners attended to the tones than when they were told to ignore them (Snyder et al., 2006). However, other studies have found that attention may not always be needed for streaming. In a mismatch negativity (MMN) study (Sussman et al., 2002), for instance, listeners heard task-irrelevant tones organized in an XOOXOO pattern, where X and O tones differed in frequency, while their task was to respond to occasional changes in the amplitude of a simultaneously presented continuous white noise signal. Most of the X tones were presented at a consistent amplitude level, but occasionally, one of the X tones was presented at a slightly louder amplitude. The O tones, however, always varied in amplitude. In this way, the X tone with the deviant amplitude would only be registered as a deviant—and the MMN would only be elicited—if listeners had segregated the X tones from the O tones. Critically, the MMN for the deviant X tone was elicited under conditions that would be expected to favor streaming, such as when there was a large frequency separation between the X and O tones or when the X deviant occurred relatively late in the stimulus train (providing enough time for the buildup of streaming), even though attention was not directed to the tones. Overall, extant data suggest that attention facilitates streaming in some situations, particularly when other cues are ambiguous, but attention may not always be necessary for streaming (Shinn-Cunningham et al., 2017).

These foundational principles of auditory streaming have important consequences for considering how and why listeners are hindered by talker variability. To foreshadow the points that will be made in the next section, the speech of a target talker likely has strong coherence among its perceptual features (such as pitch, location and timbre), as components from different sources are unlikely to covary tightly; this should support perceptual grouping of auditory information, allowing for a buildup of streaming over time (Shamma et al., 2011). The core tenet of the auditory attention hypothesis of multitalker processing costs is that when a talker’s speech is interrupted by speech from a different talker (a disruption that might be made salient by discontinuities in perceptual features such as fundamental frequency), streaming is reset, disrupting top-down attention.

### Attending to a target talker

Most relevant to the issue of multitalker processing costs is a set of auditory streaming studies focusing specifically on the challenges involved with attending to a target talker in the presence of competing background talkers. Such studies have revealed a variety of factors that can influence a listener’s ability to stream a target talker’s speech, even after accounting for individual differences (such as hearing impairment) between listeners (Bronkhorst, 2015). For example, the perceived spatial locations of target and masker talkers can influence the intelligibility of a target talker (Ericson et al., 2004; Freyman et al., 2001). Additionally, listeners are worse at identifying a target talker’s speech when a masker phrase is spoken by a same-sex talker compared with an opposite-sex talker (Brungart, 2001). Nonetheless, when presented with a mix of two voices, listeners are generally successful in tracking a cued voice over time, with better performance when the two voices are well separated in acoustic feature (e.g., F0 × F1 × F2) space and worse performance when they are acoustically close to each other (Woods & McDermott, 2015). Furthermore, the ability to successfully track a cued voice is associated with heightened fine-grained acoustic processing of the cued voice (Woods & McDermott, 2015).

When streaming is difficult, continuity in the features of an auditory object can be especially helpful for promoting the buildup of auditory selective attention over time (e.g., Best et al., 2008; Bressler et al., 2014). This is well illustrated by a study in which listeners were asked to attend to one of five simultaneously presented strings of spoken digits, with the target digit at each time point indicated via an LED light illuminated on one of five loudspeakers (Best et al., 2008). Listeners exhibited the greatest accuracy when the spatial location of the target digits was constant over time, indicating that continuity of spatial location made it easier to selectively attend to the relevant auditory information. Notably, listeners showed even greater accuracy for digits that occurred later in the target sequence, consistent with the proposal that selective attention accrues over time. Moreover, this buildup of selective attention was further heightened when all target stimuli were produced by a single talker. That is, continuity in one perceptual feature (e.g., talker) enhanced the benefit of continuity in another perceptual feature (e.g., spatial location). Taken together, these results indicate that the continuity of multiple perceptual features can make it easier to selectively attend to a target source, with streaming building over time.

Strikingly, research suggests that continuity of talker information facilitates the buildup of auditory attention in a fairly automatic fashion. In another study of auditory selective attention, listeners were presented with a string of target digits against a background of unintelligible, time-reversed speech (Bressler et al., 2014). In that study, listeners were more likely to correctly identify a target digit if they had correctly identified the preceding target digit, consistent with the proposal that auditory attention builds up over time. However, the benefit of having gotten the previous digit correct was larger when the target voice was constant between digits compared with when it varied. Critically, this same pattern of results was observed regardless of whether listeners knew in advance that the voice would change from digit to digit, suggesting that the benefit of a constant talker arises automatically (i.e., in a bottom-up fashion) rather than due to a listener’s predictions about when voice information will be continuous.

However, just as continuity of target features can facilitate the buildup of attention, stimulus-level discontinuities can disrupt attention (Maddox & Shinn-Cunningham, 2012). For instance, if listeners are tasked with directing their attention toward a syllable stream in a target spatial location, a discontinuity in talker information (such that the voice that was previously producing speech in the target spatial location is now producing speech in a distractor spatial location) can result in impaired behavioral processing of the target stream (Mehraei et al., 2018). Research has linked these stimulus-driven disruptions in attention with reductions in alpha oscillatory power, an electrophysiological signature of an individual’s ability to inhibit task-irrelevant information (Mehraei et al., 2018).

Still, while attention can be influenced by salient stimulus-level features, high-level knowledge and task goals can also guide auditory attention in a top-down fashion. For instance, lexical knowledge can shape whether a repeated stimulus, such as *stone* or the nonword *stome, is perceived as an integrated stream or whether the stimulus is segregated into two streams (Billig et al., 2013). For instance, the /s/ in *stome is relatively likely to be segregated, resulting in a stream that consists of the repeated word *dome* (note that the /t/ in *stome becomes a /d/ when segregated from the preceding /s/ because of lack of aspiration), while the /s/ in *stone* is less likely to be segregated into separate /s/ and *dohne streams. Additionally, listeners can flexibly direct their attention toward the pitch or location of a stimulus depending on task demands, often being able to ignore salient but irrelevant bottom-up changes (Maddox & Shinn-Cunningham, 2012). For this reason, auditory selective attention is best thought of as being shaped by interactions between bottom-up salience and top-down biases (Shinn-Cunningham, 2008).

### An auditory attention view of multitalker processing costs

An auditory attention account might provide an alternative explanation for the emergence of multitalker processing costs (Lim et al., 2019). In brief, the logic is that even when there are no competing streams to contend with, a change in talker can interrupt streaming and trigger an automatic, stimulus-driven reorienting of attention to the new auditory stream. This disruption of attention makes it harder to process the incoming speech signal and therefore elicits multitalker processing costs.

Whether this is a valid application of the concepts from the streaming literature could reasonably be debated. As traditionally defined, the cocktail party problem refers to a situation where multiple talkers are speaking at once, and listeners must segregate the target talker from a background signal. Studies examining multitalker processing costs consider a slightly different situation, in which one word is spoken by a time but consecutive words might be spoken by different talkers. One possibility is that multitalker processing costs do involve disruptions to attention (at least in part), but that rather than reflecting a disruption in streaming, they simply reflect an impairment in cognitive processing following a surprising event; this possibility is motivated by data showing that individuals exhibit motor slowing after unexpected events, with this effect thought to be driven by a fronto-basal ganglia network (Wessel & Aron, 2017). For the purposes of this review, I consider the auditory attention hypothesis of multitalker processing costs as described in the literature and defer to future work the question of whether this is an appropriate application of the literature on auditory streaming.

The idea that multitalker processing costs might instead emerge through an auditory attentional mechanism was suggested by Lim et al. (2019), who found that listeners were both slower and less accurate at recalling strings of digits that had been produced by multiple talkers compared with digit strings where all items were produced by a single talker; this multitalker processing cost was most strongly elicited when digits were presented with a short interstimulus interval. Historically, similar findings have been interpreted as consistent with the idea that additional cognitive resources are needed for processing talker variability. For instance, Martin et al. (1989) found that listeners showed poorer recall of spoken words (specifically, words that occurred relatively early in study lists) when items were spoken by multiple talkers compared with when they were spoken by a single talker. Martin et al. argued that when listeners hear multiple talkers during encoding, they must use cognitive resources to accommodate the talker variability; the cognitive cost of doing so is worse encoding of—and ultimately, poorer recall of—items in multitalker lists than items in single-talker lists. However, Lim et al. noted that these findings might also be explained by appealing to auditory attention; in particular, when perceptual discontinuities (such as talker changes or silent gaps) disrupt auditory attention, it should be harder to process the auditory signal and therefore harder to encode it. Such a view would explain both the relatively poor performance on mixed-talker lists as well as the interaction with interdigit delay.

Sensitivity to perceptual discontinuities was also observed in a subsequent follow-up study (Lim et al., 2021), which used a similar paradigm as the Lim et al. (2019) study but also involved the collection of electrophysiological and pupillometry data. In addition to observing multitalker processing costs, the authors found that mixed-talker trials were associated with a larger P3a response as compared with single-talker trials; the P3a is an event-related potential associated with the involuntarily redirection of attention to a deviant stimulus (Comerchero & Polich, 1998). Furthermore, the authors found that pupil size was sensitive to abrupt changes in stimulus properties; specifically, they observed an increase in pupil size for mixed-talker trials (compared with single-talker trials) when digits were presented at short interstimulus intervals, but not with long interstimulus intervals. Both the P3a and pupil-size data indicate physiological sensitivity to stimulus-level disruptions in auditory attention. Finally, Lim et al. found that single-talker trials were associated with an increase in alpha oscillatory power compared with mixed-talker trials; because alpha oscillations are associated with demands on attentional control, the increased alpha power in single-talker trials might reflect the increased buildup of selective attention associated with the single-talker trials, whereas the reduced alpha oscillations in mixed-talker trials might reflect disruptions in auditory selective attention.

Additional evidence in favor of an auditory attention mechanism comes from a series of experiments by Choi and Perrachione (2019). In their study, listeners performed a speeded classification task with two alternatives that were close in acoustic space (*boot* and *boat*). The authors found that the perceptual cost associated with a talker change was smallest when the target word was preceded by a long carrier phrase from the new talker (*I owe you a . . .*, approximately 600 ms), intermediate when the target was preceded by a short carrier phrase (*It’s a . . .*, approximately 300 ms), and largest when there was no preceding carrier phrase. A follow-up experiment showed that this effect was not due to the degree of phonetic information in the carrier phrase; the authors observed equal processing penalties between two carrier phrases that differed in phonetic content but were equated in length (*I owe you a . . .* vs. *Aaaa . . .*, both approximately 600 ms). This result can be explained by the auditory attention view: Selective attention to the new talker builds up over the course of the carrier phrase, thereby reducing the impact of the talker change by the time the target word is encountered. In a third and final experiment, Choi and Perrachione provided listeners with a short carrier phrase (*A . . .*, approximately 200 ms) prior to the target word but manipulated whether there was a short delay (roughly 400 ms) between the carrier and the target. Under the auditory attention view, a stimulus discontinuity should disrupt auditory attention, making multitalker processing costs larger than when there is no discontinuity. Indeed, the authors observed larger multitalker processing costs when there was a gap between the carrier phrase and the target stimulus than when there was not, consistent with the predictions of the auditory attention account. Taken together, the results of Choi and Perrachione’s study provide evidence that multitalker processing costs may be at least partly attributable to auditory attention.

## What drives multitalker processing costs?

### Adjudicating between contextual tuning and auditory attention accounts

Understanding the mechanism through which multitalker processing costs emerge—that is, whether they arise through a resource-demanding mapping adjustment process or automatically through disruptions in auditory attention—is critical for understanding how to interpret data from the myriad studies that have observed multitalker processing costs. By clarifying the underlying mechanism, we can better understand the processes involved in accommodating talker variability.

However, a challenge in evaluating the contextual tuning and auditory attention accounts is that much of the data that are consistent with one view may not necessarily be inconsistent with the other. Consider again the findings of Choi and Perrachione (2019). While an auditory attention account makes clear predictions about how multitalker processing costs should be affected by the length of a carrier phrase and/or stimulus discontinuities, the contextual tuning view does not make any predictions about how timing should influence multitalker processing costs; the accounts differ in their scope, so evidence in favor of one view may not necessarily constitute evidence against the other. More generally, a variety of data regarding multitalker processing costs can be explained under either view, as summarized in Table 1.

Strikingly, some recent data suggest that both auditory attention and contextual tuning mechanisms may underlie the emergence of multitalker processing costs (Choi et al., 2022). Recall that a previous study (Choi & Perrachione, 2019) found that multitalker processing costs are smaller if the new talker produces a carrier phrase prior to the target word, with greater attenuation observed for longer carrier phrases, and that this finding is readily explained by the auditory attention view. Choi et al. reasoned that under the auditory attention view, there must be some length of carrier phrase at which the multitalker processing effect would disappear. That is, with a sufficiently long carrier phrase, selective attention should build up enough that a listener’s response in the mixed-talker condition would be just as fast as their response in the single-talker condition. The authors used a simple carrier phrase (the vowel /^/ preceding *boat* or *boot*) and found that the multitalker processing cost was largest for 0-ms carrier phrases (that is, no carrier phrase), intermediate for 300-ms carrier phrases, and smallest for 600-ms carrier phrases, consistent with the previous study. However, there was no further reduction in the size of the multitalker processing cost with carrier phrases of longer durations (900 ms, 1,200 ms, 1,500 ms). Critically, this result does not reflect a limit on how quickly participants can respond overall; even for the longest carrier phrase, target responses were always significantly faster in the single-talker condition than in the mixed-talker condition. The authors interpreted this piecewise set of results—monotonic decreases in the size of multitalker processing costs for increasingly longer carrier phrases up to 600 ms, but no change in the size of the processing penalty with carrier phrases longer than 600 ms—as evidence for a dual-mechanism system. Specifically, they argued that multitalker processing costs are driven by stimulus-level discontinuities over short time scales (that is, attributable to an auditory attention mechanism) and driven by recomputing the mapping between acoustic information and perceptual categories over a longer time scale (consistent with a contextual tuning account).

The results from Choi et al. (2022) offer a compelling potential synthesis of the extant literature on multitalker processing costs and also generate testable predictions for future work. For instance, though a previous study found that talker familiarity did not influence the size of multitalker processing costs (Magnuson et al., 2021; see Table 1), the suggestion that the contextual tuning process operates over a relatively long time scale makes the prediction that a benefit of talker familiarity (i.e., knowing the appropriate acoustics-to-phonetics mapping in advance) may only emerge if listeners first hear a relatively long (≥600 ms) carrier phrase. This hypothesis is also consistent with the discussion of Magnuson et al. (2021), who hypothesized that listeners may have to perform some degree of perceptual analysis on the speech signal in order to recognize the talker (whether implicitly or explicitly) before they can select the appropriate acoustic-to-phonetic mapping. On this account, the processing of a talker’s voice would proceed in parallel with phonetic processing, but phonetic processing would also be contingent on the output of the talker recognition process (a “parallel-contingent” relationship; Mullennix & Pisoni, 1990; Turvey, 1973). A relatively long carrier phrase from a familiar talker may provide listeners with enough time to access the appropriate acoustic-to-phonetic mapping, reducing subsequent processing costs. More generally, the suggestion that multitalker processing costs are attributable to both a contextual tuning process and disruptions in auditory attention is a tantalizing one, and future work will be needed to further evaluate this hypothesis.

### Factors governing the emergence of multitalker processing accounts

The overall goal of studying multitalker processing costs is to better understand the underlying mechanisms that support speech perception, particularly with regard to the perceptual accommodation of talker variability. In evaluating the data on multitalker processing costs, then, it is important to consider the extent to which studies that have examined multitalker processing costs actually tap into speech perception, rather than simply revealing epiphenomena. To this end, it can be informative to review the circumstances under which multitalker processing costs have not emerged.

Here, it is important to be careful to distinguish between situations where multitalker processing costs may fail to emerge due to the idiosyncrasies of individual experiments versus representing a general trend. For example, as discussed in the section titled “A Contextual Tuning View of Multitalker Processing Costs,” a recent study (Luthra et al., 2021) failed to replicate the previous finding that expectations influence the emergence of multitalker processing costs (Magnuson & Nusbaum, 2007). However, it was not just that the critical interaction between expectations and talker variability was not observed; rather, an effect of talker variability was not observed at all. Such a result might simply mean that listeners had trouble mapping a subtle (10-Hz) acoustic difference onto a talker difference, which may reflect the relatively low-quality speech synthesis methods used in both the Magnuson and Nusbaum (2007) study as well as the recent replication attempt. Thus, it would be premature to conclude, for instance, that effects of talker variability are generally weak. Instead, it would be informative to test whether such costs might be observed (and possibly modulated by high-level expectations) in a study using more naturalistic stimuli before drawing conclusions about the conditions under which multitalker processing costs emerge and/or are extinguished.

On the other hand, some work suggests that the emergence of multitalker processing costs might in fact be tied to task demands, at least to a certain degree. To illustrate, consider that much of the extant data on multitalker processing costs comes from studies employing the speeded monitoring paradigm (Heald & Nusbaum, 2014; Magnuson & Nusbaum, 2007; Nusbaum & Morin, 1992; Wong et al., 2004). In this paradigm, listeners hear a stream of auditory stimuli (e.g., *dime, priest, lash, ball, gnash, knife*) and have to respond whenever a visually indicated target (e.g., BALL) is produced; in blocked trials, all items in a stream are produced by a single talker, and in mixed trials, stimuli are produced by varying talkers. As traditionally implemented, a critical difference between conditions is that in blocked trials, listeners only need to monitor for a single unique token (e.g., a single acoustic exemplar of *ball*); however, mixed trials include target items produced by two talkers, so listeners must monitor for two unique tokens. That is, in the typical monitoring paradigm, blocked and mixed trials differ not only in whether there is talker variability but also in how many tokens a listener must monitor for. Critically, recent work shows that multitalker processing costs are eliminated if all the target items in a mixed trial are produced by a single talker (Saltzman et al., 2021; see Table 1); note that these mixed-talker trials still contain word-to-word talker variability, but only the speech of one talker requires a response. Such a result suggests that multitalker processing costs in the speeded monitoring paradigm may be due to differential demands on monitoring between conditions and not due to the talker variability in and of itself, at least in part.

Additionally, experimental work has highlighted conditions where talker changes have no apparent effect on speech processing. For instance, Newman (2016) conducted a semantic priming study in which auditory primes (e.g., *kidney*) preceded semantically related visual targets (e.g., BEAN); of interest, Newman observed equivalent amounts of priming when there was a gender change partway through the prime (e.g., a male talker producing *kid*, a female talker producing *knee*) compared with when a single talker produced the full prime word. That is, there was no measurable effect of introducing talker variability. Such a result might suggest that multitalker processing costs are relatively weak and/or that talker changes may not influence lexical access. Alternatively, the data may speak to the limitations of the semantic priming paradigm for measuring processing penalties associated with talker changes. Consider that multitalker processing costs typically manifest as slower, but not less accurate, word recognition. The semantic priming paradigm may not be sensitive to such a difference; if activation of the prime word is slightly slower in the presence of a talker change but the prime word is still activated to the same degree by the time the target word is presented, then equivalent amounts of priming should be observed.

More generally, while the majority of studies demonstrating multitalker processing costs have utilized either the speeded monitoring or speeded identification paradigms, multitalker processing costs have been observed in myriad tasks that tap into speech perception, including vowel identification (Verbrugge et al., 1976), word-in-noise identification (Mullennix et al., 1989), word repetition (Mullennix et al., 1989), and word recall (Goldinger et al., 1991; Martin et al., 1989). The consistent pattern of results across these tasks suggests that these findings likely reflect a core aspect of speech perception, rather than simply tapping into a quirk of some singular paradigm. Nevertheless, the extant data do not unequivocally identify the point(s) in the processing stream at which talker variability hinders speech perception. To do so, it is necessary to work toward a model of speech perception that integrates auditory attention.

## Toward an integrated account of auditory attention and talker accommodation

### Integrating across levels of analysis

At a macroscopic level, our goal is to move toward a process account of how listeners contend with talker variability—that is, an account that is positioned at an algorithmic level of analysis (Marr, 1982). Doing so will require integrating across multiple levels of analysis, considering both the high-level problem of accommodating talker variability (a computational-level approach) and the underlying biological mechanisms through which such a problem might be solved (an implementational-level approach). While it can be useful (and certainly more tractable) to consider each of these levels of analysis separately, they all pose important constraints on each other, and considering the interactions between levels of analysis is ultimately important for moving toward an understanding of how auditory attention and talker accommodation mechanisms might interact.

To illustrate, a number of mathematical frameworks have considered the issue of between-talker variability at the computational level of analysis. The Bayesian belief-updating model (Kleinschmidt & Jaeger, 2015), for instance, provides a mathematical formalism for how listeners might adjust their (implicit) beliefs about which acoustics correspond to which perceptual categories in a talker-specific fashion. Additionally, classifiers that use cues that are scaled relative to the mean values for each talker (referred to as the computing cues relative to expectations, or C-CuRE, approach) have been shown to provide a better fit to human perceptual data compared with models using raw acoustic values (McMurray & Jongman, 2011; see also Crinnion et al., 2020). Because they are positioned at the computational level of analysis, these approaches do not address how such a computation might be achieved algorithmically. Still, it is useful to consider such a question. Are such computations compatible with passive perceptual models, or do they require active control mechanisms (and therefore cognitive resources) in order to be achieved? Might the belief-updater and C-CuRE frameworks be functionally equivalent to the contextual tuning theory, and if not, how might they differ? If these mathematical goals cannot be achieved using the contextual tuning approach, how might we modify the contextual tuning framework accordingly?

Similarly, neurobiological data (which consider the issue of talker variability at the implementational level) may be useful for adjudicating between candidate algorithms. Some informative EEG data come from a study by Uddin et al. (2020), in which participants passively listened to auditory sentences, some of which were produced by a single talker and some of which involved a talker change on the final word. The researchers predicted that talker variability might influence the event-related potential elicited by the final word, and in particular, that talker changes would affect the amplitude of N1 and P2 components, both of which have been linked to attentional processing. Unfortunately, clear N1 and P2 signals were not elicited, precluding an analysis of how talker variability might influence component amplitude; the authors hypothesized that this might have been attributable to the use of fluent sentences without silent gaps, rather than presenting words in isolation. However, Uddin et al. did observe significant effects of talker variability in the N1 and P2 time windows, even if the scalp topography differed from typical N1-P2 responses. Strikingly, the size of the effect in the N1 window significantly correlated with subject-wise performance on an auditory working memory task. Additionally, a source analysis found a significant difference in the variance explained by temporal cortex sources in the single-talker and multiple-talker conditions. On the basis of these results, Uddin et al. reasoned that contending with talker variability may be accomplished in two stages: an early stage that depends on working memory resources, followed by a speech analysis stage supported by temporal sources. Such a result is also potentially consistent with the proposal that the accommodation of talker variability is supported by a two-stage mechanism: an early auditory attention mechanism and a later contextual tuning mechanism (Choi et al., 2022).

The results from Uddin et al. (2020) highlight some of the advantages of investigating neurobiological mechanisms while also illustrating some of the limitations. While passive experiments are often necessary for straightforward interpretation of the EEG signal, the lack of behavioral data preclude an evaluation of whether multitalker processing costs emerge behaviorally with this particular paradigm. Additionally, the authors limited their source analysis to two regions that had been implicated by a previous fMRI study (Wong et al., 2004): a superior parietal lobule source and a superior/middle temporal gyrus source. However, it might be informative to also consider other possible sources as well. As described above, it is possible that early responses to talker variability reflect not a disruption in streaming but rather the engagement of a fronto-basal ganglia suppression network in response to a surprising event (Wessel & Aron, 2017). Neurobiological data could be highly informative for adjudicating between a global suppression mechanism and an auditory streaming one. Thus, as we look to develop an algorithmic account of how listeners contend with talker variability, it will be useful to consider neurobiological data, while also being aware of the limitations of such data.

### Beyond talker variability

Talker variability constitutes just one of the myriad types of acoustic variability with which a listener must contend. Listeners must also contend with acoustic variability as a function of factors like speaking rate (Miller, 1981), phonetic context (Liberman et al., 1967), and within-talker variability (Kleinschmidt & Jaeger, 2015). An important consideration for future work is to evaluate the extent to which the challenges involved in contending with talker variability may also emerge with other forms of variability.

On the one hand, there may be reason to think that talker variability is “special.” The speech signal carries rich information about talker identity (Foulkes & Hay, 2015), and listeners are highly sensitive to such information, readily recognizing what phonetic variation (Kraljic & Samuel, 2007; Theodore & Miller, 2010) and semantic content (King & Sumner, 2015; Van Berkum et al., 2008) are typical of an individual talker (or group of talkers). Results from computational modeling studies suggest that it is helpful to condition speech perception on talker information specifically (Kleinschmidt, 2019), owing to the structured variation in how different individuals (or sets of individuals) produce their speech sounds (Allen et al., 2003; Hillenbrand et al., 1995; Newman et al., 2001). Additionally, neurobiological data suggest that the integration of talker information and phonetic detail may be supported by interactions between a right-lateralized neural system for vocal identity processing and a left-lateralized system for speech perception (Luthra, 2021; Luthra et al., 2023), whereas the processing of rate variability, for instance, is largely supported by left hemisphere regions involved in speech perception (Ruff et al., 2002; but see Poldrack et al., 2001). Taken together, then, extant data suggest that the mechanisms supporting the accommodation of talker variability may be distinct from other types of acoustic variability.

On the other hand, variability-related processing costs have been observed for other forms of acoustic variability, including processing costs related to variation in speaking rate (Sommers & Barcroft, 2006; Sommers et al., 1994), speaking style (Sommers & Barcroft, 2006), and within-talker token variability (Drown & Theodore, 2020; Kapadia et al., 2023; Uchanski & Braida, 1998), though not for phonetically irrelevant variation, such as variation in stimulus amplitude (Sommers & Barcroft, 2006). Additionally, there is some evidence that nonhuman animals are also able to perform some form of “talker” normalization, though it remains to be seen whether the same mechanisms underlie this process in humans and nonhuman animals (Kriengwatana et al., 2014). Overall, these data raise the possibility that talker variation is just a specific example of acoustic variability, such that developing an algorithmic account of how listeners contend with talker variability may require us to consider other forms of acoustic variability that contribute to phonetic variation.

How might processing costs related to acoustic (but not necessarily talker) variability be explained by contextual tuning and/or auditory attention accounts? Under a contextual tuning framework, a higher degree of acoustic variability might induce uncertainty as to whether the current mapping is appropriate, even in the absence of an overt talker change; as schematized in Fig. 1, this would initiate the resource-demanding mapping computation stage. However, it is less clear how within-talker variability would lead to a disruption of auditory streaming, particularly if other relevant cues for streaming (e.g., pitch, timbre, location) are relatively stable; as reviewed above, continuity in these features facilitates streaming over time (Best et al., 2008; Shamma et al., 2011), meaning that the degree of within-talker variability would have to be fairly substantial to disrupt streaming. As such, it may be that within-talker variability imposes a processing cost specifically by introducing uncertainty as to whether the current acoustic-to-phonetic mapping is appropriate, whereas between-talker variability may impose a processing cost because the current mapping is inappropriate and/or because of disruptions to auditory streaming. As we work to develop richer models of talker accommodation, it will be important to consider the extent to which our models may instead reflect the accommodation of acoustic variability more generally.

Additionally, as we begin to consider other forms of acoustic variability, it may be worth considering the extent to which the models we develop are specific to speech perception or might be applied to auditory processing more generally. As currently instantiated, the contextual tuning model as illustrated in Fig. 1 is specific to the accommodation of talker variability in speech perception, whereas the principles of auditory streaming apply to both speech and nonspeech stimuli. Might a model of talker accommodation in speech perception be better constructed as a model of accommodation of acoustic structure in auditory processing, allowing it to also account for processing costs in nonspeech domains (e.g., processing costs in music perception related to variability in musical instruments; Shorey et al., 2022)? Such questions will be important to consider as we develop our mechanistic understanding of how listeners accommodate talker variability.

## Concluding remarks

Listeners endure processing penalties when there is variability in the identity of the talker producing speech. Such multitalker processing costs have been argued to be a consequence of a resource-demanding computation of the mapping between acoustics and phonetic categories, and they have also been argued to reflect disruptions in top-down auditory attention caused by salient stimulus-level discontinuities, among other potential explanations. Recent data (Choi et al., 2022) suggest that the processing costs associated with talker variability may be partly attributable to both contextual tuning and auditory attention mechanisms operating over different time scales. As such, future work should attempt to determine the extent to which each of these mechanisms underlies these processing costs, to clarify the precise time scales over which these processes operate, and to determine which other factors can modulate the timing of these processes.

As researchers continue to consider the issue of why listeners are hindered by talker variability, it will be important to carefully consider this issue both through the lens of auditory attention and in terms of speech perception. It is clear that both auditory attention mechanisms and speech perception mechanisms must be at play when listeners contend with talker variability; however, theoretical models of talker accommodation and auditory streaming have been formulated relatively independently, with little contact between these two literatures. Certainly, there are advantages for studying these topics in isolation—the lack of invariance problem is far more tractable when considered independently from auditory attention, and it is far more practical to study the cocktail party problem without the added complications of talker-specific phonetic variation. Furthermore, extant models of speech perception (e.g., Kleinschmidt & Jaeger, 2015; Magnuson et al., 2020; McClelland & Elman, 1986; Norris et al., 2000) and of auditory attention (Cusack et al., 2004; Holmes et al., 2021; Shamma et al., 2011) have explained a considerable amount of empirical data. Nevertheless, given that some phenomena—such as the emergence of multitalker processing costs—may be driven by both speech perception and auditory attention mechanisms, it is useful to attempt to consider how these mechanisms might interact.

What might a theory of talker accommodation that includes a role for auditory attention look like? One possibility is that when listeners encounter a change in talker, a stimulus-level discontinuity (e.g., in F0) disrupts auditory attention, resetting streaming. If attention to the target talker is necessary for (or at least facilitates) the buildup of streaming, then directing attentional resources toward the buildup of streaming should limit the cognitive resources that are available for adjusting the mapping between acoustics and phonetics. In this way, then, auditory streaming and adjustments to the acoustics-to-phonetics mapping may be interrelated processes, and it could be that either or both processes underlie the emergence of multitalker processing costs specifically. However, there are several key questions that will need to be tested empirically as scientists work to develop a model of talker accommodation that includes a role for auditory attention. For example, to what degree does auditory attention precede speech perception (i.e., are these serial processes), and to what degree do these processes operate in parallel? Are they relatively independent processes, or do they inform each other?

Overall, the studies reviewed here highlight the need to better integrate auditory attention into theories of speech perception. Beyond clarifying the nature of multitalker processing costs, an integrated theory could lead to a more thorough understanding of the interactions between auditory attention and speech perception, allowing for a more precise characterization of how listeners understand the speech signal. This will be particularly informative for studying speech perception in ecological, real-world listening conditions (e.g., a crowded café), where listeners must not only map from the acoustic signal to perceptual categories but also selectively attend to one source while filtering out other auditory sources.

## Figures and Tables

**Table 1:** Several findings regarding the emergence of multitalker processing costs are explainable under the contextual tuning theory as well as the auditory attention view

Study and paradigm	Key Finding	Explanation under contextual tuning theory	Explanation under auditory attention view

Mullennix et al. (1989) *(1) Word identification in noise; (2) word repetition*	Processing costs observed across multiple word recognition tasks	Cognitive resources needed to recompute mapping between acoustics and phonetic categories	Disruption in auditory attention makes it harder to process words following a talker change, leading to slower responses
Heald and Nusbaum (2014) *Speeded word monitoring*	Processing costs are larger when listeners have both auditory and visual input, compared with auditory input alone	Visual information strengthens *expectation* of talker change (see Magnuson & Nusbaum, (2007)	Visual information provides high-level cue for second auditory object, disrupting top-down auditory attention
Choi et al. (2018) *Speeded word identification (2AFC)*	Processing costs are larger when vowels are highly confusable in F1 × F2 space *(boot, boat)* than if alternatives are dissimilar *(beet, boat)*	The closer that two categories are to each other in acoustic space, the more cognitive resources are needed	Talker change disrupts attention, impairing perceptual processing. This manifests as longer processing when alternatives are relatively close in F1 × F2 space
Lim et al. (2019) *Digit recall*	Processing costs are increased when digits are presented at short ISI	Since cognitive resources are needed for mapping adjustments, fewer resources are available for encoding/rehearsal of digits	Perceptual discontinuities (such as talker changes or silent gaps) disrupt auditory attention, impacting encoding of digits
Kapadia and Perrachione (2020) *Speeded word identification (2AFC)*	Processing costs are not affected by the number of talkers producing stimuli in mixed-talker blocks (2, 4, 8 or 16)	Any talker change requires listeners to adjust acoustic-to-phonetic mapping, eliciting a processing cost	Any talker change induces a stimulus-level discontinuity, impairing top-down attention and eliciting a processing cost
Stilp and Theodore (2020) *Speeded word identification (2AFC)*	Greater F0 variability between talkers is associated with larger processing costs; processing costs are also larger when talkers vary randomly compared with when trials are blocked by talker	Larger F0 change provides stronger expectation of talker change and thus larger costs; increasing number of talker changes leads to increased processing cost	Increasing the size (in terms of F0) or frequency of talker changes leads to larger or more frequent stimulus-level discontinuities, thus inducing larger processing costs
Saltzman et al. (2021) *Speeded word monitoring*	Processing costs are eliminated if all the targets on mixed-talker trials are spoken by a single talker	Processing costs are only observed when listeners adjust acoustics-to-phonetic mapping for purposes of making a behavioral response	When only one talker is behaviorally relevant, top-down allows listeners to “block out” distractor talker, eliminating or attenuating multitalker processing cost
Magnuson et al. (2021) *Speeded monitoring*	Talker familiarity does not influence size of multitalker processing cost, though familiarity leads to improved recognition of speech in noise	The process of *adjusting* the acoustics-to-phonetics mapping must be completed after every talker switch, even if the mapping need not be *computed*	Any talker change disrupts attention, eliciting a talker processing cost

## Footnotes

1 Under the view that the accommodation of talker variability implies *destructive abstraction* (i.e., that surface details are discarded when acoustics are translated to abstract phonetic categories), the myriad findings showing that listeners can maintain talker-specific details in memory (Church & Schacter, 1994; Goldinger, 1996; Luthra et al., 2018; Mattys & Liss, 2008; McLennan & Luce, 2005; Schacter & Church, 1992; Theodore et al., 2015) should be taken as evidence against the proposal of normalization—because, it is argued, such findings suggest that perceptual categories are not abstract to begin with. Indeed, based on such results, some researchers have argued that listeners store auditory traces of every speech encounter and that speech perception involves comparing the auditory signal to these stored episodes (Goldinger, 1998). A thorough discussion of the nature of perceptual categories—specifically, whether they are abstract or episodic—is beyond the scope of this paper, and the interested reader is directed to a recent review by Pierrehumbert (2016). For the purposes of the current review, I assume that phonological representations are relatively abstract, and I make two notes as I do so. First, I note the idea advanced by Pierrehumbert that any representation, by its nature, entails some degree of abstraction. Second, I highlight that the question of whether listeners can retain talker information in memory is, in principle, distinct from the question of whether listeners map speech onto abstract perceptual categories, as listeners might have multiple representations of speech sounds that differ in their level of abstraction (Magnuson & Nusbaum, 2007; Weatherholtz & Jaeger, 2016).
